# Stochastic Vehicle Load Simulation for Small- and Medium-Span Bridges Based on Weigh-in-Motion Monitoring

**DOI:** 10.3390/s26051681

**Published:** 2026-03-06

**Authors:** Ping Fan, Gang Wu, Zhenwei Zhou, Bitao Wu, Xuzheng Liu

**Affiliations:** 1 Research Institute of Highway, Ministry of Transport, Beijing 100088, China; p.fan@rioh.cn; 2 School of Civil Engineering & Architecture, East China Jiao Tong University, Nanchang 330013, China; wubitao@yeah.net (B.W.); urbwolf@126.com (X.L.)

**Keywords:** stochastic vehicle load, bridge health monitoring, weigh in motion, small- and medium-span bridges, Monte Carlo

## Abstract

Vehicle loads constitute the dominant source of dynamic excitation for small- and medium-span bridges, exerting a critical influence on bridge safety and service performance. However, vehicle load characteristics exhibit pronounced temporal variability and strong regional heterogeneity, which poses challenges for accurately characterizing the in-service loading conditions of bridges in specific regions using conventional dynamic load models. Therefore, this study focuses on the actual operational characteristics of vehicles on the Lieshihe bridge and the effects of vehicle loads and proposes a stochastic vehicle load simulation method based on the Monte Carlo sampling technique and weigh-in-motion (WIM) measured data. Initially, the recorded vehicle data are classified into representative vehicle models, and statistical analyses are conducted to characterize lane-dependent traffic flow variations and the occurrence patterns of vehicle overloading. Subsequently, axle number and axle spacing are selected as the core indicators for vehicle classification, based on which vehicles are categorized into five representative vehicle types. The changing patterns of axle load, vehicle weight, vehicle speed, etc., for each vehicle type are studied, and corresponding probability density distribution models are established to describe the stochastic nature of vehicle characteristics. Finally, using the Monte Carlo method combined with important attributes of vehicle flows, a stochastic vehicle load model is established based on the spatial–temporal characteristics. The results demonstrate that the vehicle weight on the bridge exhibits a Gaussian mixture distribution with multi-peaks, characterized by similar peak magnitudes but markedly different occurrence frequencies; axle load shows a single-peak distribution of Gaussian distribution with small differences in peak values and frequencies.

## 1. Introduction

Vehicles represent the primary variable loads on in-service bridge structures, significantly influencing the construction and maintenance of modern transportation infrastructure [1,2,3]. With the rapid development of the automotive industry and transportation, a large number of heavy-duty vehicles have emerged, and overloading incidents occur frequently. The vehicle types on in-service bridges exhibit characteristics such as randomness, spatial–temporal, and regional differences. Existing bridge structures are inadequately represented by the vehicle loading models specified in design standards, failing to accurately depict the actual load conditions during operation [4,5,6]. The impact of vehicle load on medium- and small-span bridges disproportionately contributes to the overall design load effects. Notably, the detrimental effects of vehicle overloading are substantially more pronounced for small- and medium-span bridges when compared to long-span bridges [7,8]. Therefore, accurately understanding the vehicle load effects of in-service small- and medium-span bridges and accurately establishing a probability model of random vehicle effects are crucial to ensuring the safe operation of bridges.

With the development of electromechanical technology, weigh-in-motion (WIM) systems can dynamically record vehicle parameters such as speed, axle load, weight, and axle spacing without interrupting vehicle operation and have been widely used in bridge health monitoring [9,10,11,12,13,14,15]. Utilizing WIM monitoring data, vehicle load effect calculations can be performed, and extreme value theory can be applied to establish vehicle load models and predict future vehicle load conditions. Modeling vehicle load based on the measured WIM data effectively compensates for the shortcomings of traditional theoretical models and significantly improves vehicle load representation accuracy [16,17,18]. Gu et al. [19] developed Bayesian extreme value distribution functions to describe the probability distribution of total vehicle weight extremes on the Nanjing Yangtze River Third Bridge. Meyer et al. [20] established a vehicle load model based on WIM measured data to explore the dynamic effects induced on bridges by long, multi-trailer heavy vehicles compared to smaller heavy vehicles. Tabatabai et al. [21] conducted a statistical analysis on truck-type data from WIM data and statistically evaluated the extreme load impact of different truck categories on bridges. Nowak and Rakoczy [22] found from WIM measured data that gross vehicle weights (GVWs) obey a normal distribution, but Hou et al. [23] demonstrated in subsequent research that GVWs in different lanes may obey different distributions, and GVWs may also follow Beta distributions. Long-term monitoring data involving millions of recorded vehicles have confirmed that WIM-based traffic load modeling provides substantially more realistic load representations than traditional code-based models. Li et al. [24] proposed a statistical information-based denoising diffusion implicit model based on the WIM measured data to generate spatial–temporal vehicle load data including time intervals, total weight, axle number, and corresponding lanes. The above research indicates that the vehicle load models of bridges vary significantly across different regions, leading to substantial differences in the corresponding stochastic vehicle load models. For bridges of different types in different regions, accurate representation of the actual vehicular load conditions should be achieved through statistical analysis of site-specific measured data [25,26]. This imbalance arises partly because long-span bridges are typically instrumented with monitoring systems, whereas small- and medium-span bridges often lack continuous monitoring data despite representing the majority of bridges [27,28]. Thus, most existing studies have focused on long-span bridges, whereas investigations of small- and medium-span bridges remain limited. The distribution of vehicle types on small- and medium-span bridges differs significantly from that on long-span bridges, and the structural response of small- and medium-span bridges to vehicle loads is more pronounced than that of long-span bridges. Moreover, different single and mixture probability distribution models should be introduced for different vehicle operating states to further study the distribution characteristics of statistical parameters in vehicle loads, such as vehicle type, weight, axle spacing, and speed in different lanes.

From the abovementioned information, it is evident that research on vehicle load simulation has become a hot topic in the field of bridge engineering, yielding significant achievements. However, the development of the automotive industry and transportation sector has led to changes in current vehicle parameters, such as weight, axle load, and speed, compared to the reviewed literature. Moreover, vehicle load conditions vary in different regions, with many areas experiencing a significant number of overloaded vehicles, making the distribution characteristics of vehicle load more complex. In particular, most studies currently focus on the vehicle load characteristics of large-span bridges, while the effects of vehicle load on small- and medium-span bridges are more significant, necessitating further exploration of spatiotemporal evolution aspects. In this context, this study categorizes and statistically evaluates typical vehicles to examine traffic volume temporal trends on these bridges utilizing extensive monitoring data from WIM data on a specific small- and medium-span bridge. Probability distribution models for vehicle speed, weight, and axle load of diverse vehicle types in different lanes are established. By identifying feature parameters that effectively encapsulate the overall distribution characteristics of vehicle load models, a stochastic vehicle load model is proposed. The proposed framework integrates vehicle types, lane differentiation, and spatiotemporal statistical characteristics into a Monte Carlo stochastic simulation procedure, which enhances the representativeness and transferability of the vehicle load model for small- and medium-span bridges. The efficacy of the proposed stochastic vehicle load model is validated through a comparison with measured vehicle load data on bridges.

## 2. Theoretical Background

Vehicle loads exhibit significant variability in both numerical values and spatial distribution on in-service bridges. Adopting mathematical statistics methods to determine the statistical parameters and probability distribution types of vehicle loads requires fitting the data to obtain a probability density distribution function. Research [29] has demonstrated that vehicle loads (parameters such as vehicle type, weight, axle spacing, etc.) follow common unimodal probability distributions, such as Gaussian distribution, log-normal distribution, Gamma distribution, Weibull distribution, and a Gaussian mixture distribution model.

### 2.1. Probabilistic Models of Vehicle Parameters

#### 2.1.1. Gaussian Distribution

Gaussian distribution is a very important probability distribution in mathematics and engineering. The distribution curve has characteristics of concentration, symmetry, and uniform variability, and the probability density function is given as

(1) $$f(x)=\frac{1}{\sqrt{2\pi }\sigma }e^{\frac{-{\left(x-\mu \right)}^{2}}{2\sigma^{2}}}\;\;-\infty <x<+\infty$$

where μ and σ represent the mean value and standard deviation.

#### 2.1.2. Log-Normal Distribution

Log-normal distribution refers to a random variable for which the logarithm follows a normal distribution. In other words, if ln(X) follows a normal distribution, then the distribution of X is called log-normal distribution. Its probability density function can be expressed as

(2) $$f(x)=\left\{\begin{array}{ll} \frac{1}{x\sigma \sqrt{2\pi }}e^{\frac{{\left(\ln x-\mu \right)}^{2}}{2\sigma^{2}}} & x>0\\ \;\;0 & x\leq 0 \end{array}\right.$$

#### 2.1.3. Gamma Distribution

The Gamma distribution is a continuous probability function in statistics, more general than the exponential distribution and normal distribution. It can be used to represent different practical distributions such as early failure, random failure, and wear failure. Its probability density function is as follows:

(3) $$f(x)=\left\{\begin{array}{ll} \frac{\lambda^{\alpha }x^{\alpha -1}}{\Gamma (\alpha )}e^{-\lambda x} & x\geq 0\\ \;\;\;\;0 & x<0 \end{array}\right.$$

where the Gamma distribution becomes the exponential distribution if the distribution parameter α=1.

#### 2.1.4. Weibull Distribution

Weibull distribution is the theoretical basis for reliability analysis and remaining life analysis, widely used in engineering. Also, it can easily infer its distribution parameters using probability values and is widely applied in various statistical data processing. Its probability density function can be calculated as follows:

(4) $$f(x)=\left\{\begin{array}{ll} \frac{k}{\lambda }{\left(\frac{x}{\lambda }\right)}^{k-1}e^{-{\left(x/\lambda \right)}^{k}} & x\geq 0\\ \;0 & x<0 \end{array}\right.$$

where λ and k are the scale and shape parameters. Weibull distribution is related to many distributions, such as when k = 0, it is the exponential distribution, and when k=2, it is the Rayleigh distribution function.

#### 2.1.5. Gaussian Mixture Distribution

The variability in vehicle loading conditions within WIM measured data, influenced by factors such as diverse modified vehicles, results in the manifestation of bimodal or even multimodal distribution patterns of vehicle load parameters, in addition to the unimodal distribution form. In cases of multimodal distribution, the aforementioned probability distribution function fails to meet the fitting criteria and necessitates description through a combination of multiple distribution functions. The probability density function of the Gaussian mixture distribution can be formulated as follows:

(5) $$f(x)=\sum_{i=1}^{N}p_{i}\frac{1}{\sqrt{2\pi }\sigma_{i}}e^{-\frac{{\left(1-\mu_{i}\right)}^{2}}{2\sigma_{i}^{2}}}$$

where $\sum_{i=1}^{N}p_{i}=1$, N represents the number of Gaussian distributions and $p_{i}$, $\mu_{i}$, and $\sigma_{i}$ represent the mixing weight, mean value, and standard deviation of the ith Gaussian distribution.

### 2.2. Stochastic Vehicle Load Modeling

#### 2.2.1. Monte Carlo Sampling

Relevant studies [30] have shown that there is a correlation among vehicle load parameters, such as axle loads and axle spacing. Monte Carlo-based methods [31,32] for stochastic vehicle simulation are widely adopted in practical engineering because it significantly simplifies computation while still providing realistic vehicle effect estimates. Among them, the vehicle parameters are modeled using their distributions and treated as independent random variables. Vehicle load simulation involves the depiction and modeling of the probability distributions of vehicle parameters, encompassing vehicle types, weights, axle load, and lanes. Once a probabilistic statistical model of vehicle load parameters is established, random variables following any distribution can be derived through transformation operations. Consequently, a series of uniformly distributed random numbers $\zeta_{1},\zeta_{2},\;\ldots ,\;\zeta_{n}$ within the interval [0,1] is generated, facilitating the derivation of a function representing a random variable with any distribution through mathematical transformations, and it is given as

(6) $$\eta =F^{-1}(\;)$$

where $F^{-1}(\;)$ is the inverse function of η.

#### 2.2.2. The Framework of the Stochastic Vehicle Load Model

**Figure 1:**
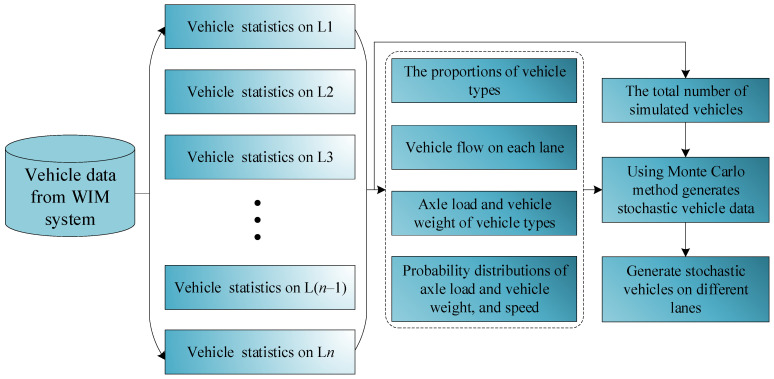
The proposed vehicle load model from WIM data based on Monte Carlo sampling method.

Figure 1 presents the framework of the proposed stochastic vehicle load model based on the Monte Carlo sampling method and mainly includes the following three steps:

(1) A comprehensive database of vehicle parameters is established based on data collected from the WIM system. A lane-specific simulation strategy is adopted, whereby statistical analyses are performed on vehicle parameter characteristics for each traffic lane on the bridge deck (e.g., Lane 1 (L1), Lane 2 (L2), etc.).
(2) The proportions of different vehicle types in each lane are quantified, and the vehicle flow rates for individual lanes are statistically analyzed. On this basis, axle load and vehicle weight parameters corresponding to different vehicle types are extracted, followed by probabilistic distributions of critical vehicle parameters, including axle load, vehicle weight, and vehicle speed.
(3) Vehicle load parameters, such as vehicle type, vehicle weight, and axle load, are treated as mutually independent and uncorrelated random variables. Based on the statistically obtained vehicle counts, the Monte Carlo sampling method is employed to generate stochastic traffic flow models for different vehicle types across individual lanes at various time periods. Consequently, the stochastic vehicle load model acting on the bridge is constructed.

## 3. Proposed Random Vehicle Flow Modeling Based on the WIM Data

### 3.1. Description of the Monitored Bridge and WIM System

**Figure 2:**
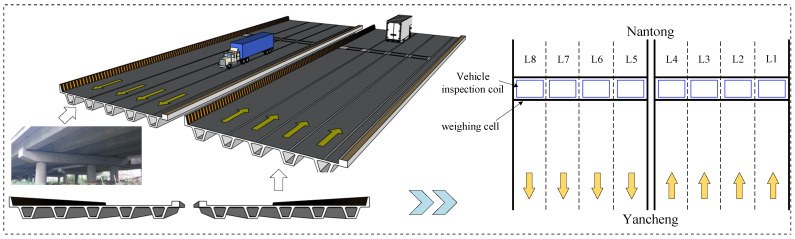
Layout of the weight-in-motion system on Lieshihe bridge.

The Lieshihe bridge, shown in Figure 2, is a representative continuous beam bridge on the Jiangsu coastal expressway in China, spanning the Lieshihe in Rugao city. The upper structure of the bridge adopts a partially prestressed concrete composite box girder with a 25 m span and a total length of 2168.20 m. A WIM system is installed at the south end of the bridge to monitor the vehicle load situation on the bridge. This WIM system installs two piezoelectric sensors and one inductive loop sensor on each lane to detect the vehicles passing through the piezoelectric sensors, which are used to calculate axle load, vehicle weight, vehicle speed, and other information. Additionally, the controller monitors the signals from the inductive loop to determine the lane information of the current passing vehicle. The bridge is divided into two sides, and the right side is from the Yancheng to Nantong direction, with the system monitoring lanes numbered L1–L4, where L1 is the emergency lane, L2 and L3 are travel lanes, and L4 is the overtaking lane. Similarly, the left side is from the Nantong to Yancheng direction, with the system monitoring lanes numbered 5–8 (L5–L8), where L8 is the emergency lane, L6 and L7 are travel lanes, and L5 is the overtaking lane. The measurement error of the WIM system is less than 10%, the measured speed range is 5 km/s to 200 km/s, the vehicle flow monitoring guarantee rate is greater than 98%, the speed measurement error is less than 1.5%, the vehicle axle spacing measurement error is less than 2%, and it can simultaneously monitor critical vehicle load parameters such as axle load, axle number, vehicle weight, and axle spacing.

### 3.2. Vehicle Classification

During the long-term operation of bridge WIM systems, a variety of factors, including inherent equipment errors, electromagnetic interference, extreme weather conditions, and human interventions, can result in anomalous measurement data. Furthermore, specific driving behaviors of vehicles passing through the weighing area can also contribute to data inaccuracies. For instance, when adjacent vehicles pass through the weighing zone at low speeds, the system may erroneously classify them as a single vehicle; conversely, vehicles with excessive lengths may be incorrectly identified as two separate vehicles. Consequently, to ensure data reliability, monitoring data that satisfy any of the following criteria, as determined based on the long-term operational history of the Lieshihe bridge, were excluded: (1) vehicle speed < 5 km/h or >200 km/h; (2) axle number < 2; (3) vehicle length > 40 m; (4) vehicle weight: <0.5 t for two-axle vehicles and <1 t for vehicles with more than two axles; (5) axle load < 0.1 t or >200 t; (6) axle spacing, the distance between axle 1 and axle 2 <1.5 m and <1 m for other axles.

**Figure 3:**
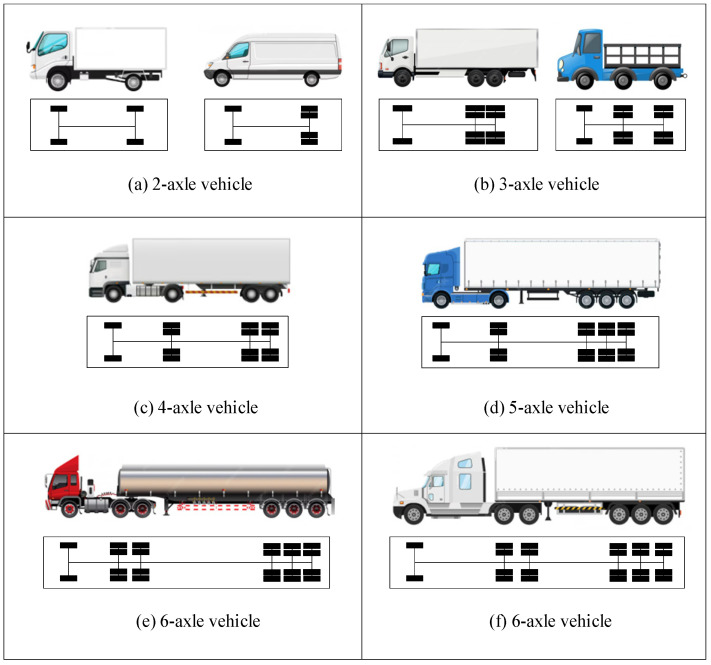
Typical vehicle types and axle configuration.

This study begins by defining the fundamental requirements for stochastic vehicle load modeling, with axle number and axle spacing selected as the primary indicators for vehicle classification. These two parameters directly influence the load distribution characteristics, thereby affecting the stress amplitudes and fatigue cycle characteristics of bridge components. The WIM data were collected from the Lieshihe bridge between May and September 2021 without interruption, which ensures that the primary vehicle loads and operational characteristics of the bridge are adequately captured. According to engineering experience and practical considerations, the following criteria were adopted to construct an effective stochastic vehicle load model: first, considering that vehicles weighing less than 1 t have a minor impact on bridge safety, only vehicles weighing more than 1 ton were considered in the WIM data; second, rare vehicle types accounting for less than 1% of the total sample and less than 5% within the same axle-number category were excluded. After this screening process, eight representative vehicles were ultimately identified and further grouped into five categories of typical vehicles, which serve as benchmark samples for bridge operational loads, as shown in Figure 3.

## 4. WIM-Based Vehicle Characteristics and Vehicle Flow Simulation

### 4.1. Vehicle Flow Statistics and Analysis

The vehicle flow serves as the total sample for vehicle load statistics, with each sub-sample that makes up the total sample being representative vehicles. A total of 2.64 million vehicles were collected based on the WIM data from May to September 2021. As lanes 1 and 8 are emergency lanes with less traffic, they are not included in the statistical scope. Table 1 presents the statistical data from L2 to L7 for each month. The results indicate that the vehicle flow crossing the Lieshihe bridge increased monthly from May to September, with a slight decrease in August and September. The reason for this decrease is mainly due to missing data in the WIM system on L4. Overall, the vehicle flow gradually increased during these months. The flow of heavy vehicles remains relatively stable, mainly traveling on L2 and L7, with a small portion on L3 and L6 and very little on L4 and L5, which accurately reflects the actual traffic situation. Additionally, the main type of vehicles crossing the bridge are 2-axle buses, accounting for the highest proportion, and primarily traveling on L3 and L5; the flow of 6-axle vehicles is also significant, mainly on L2 and L7. It is noteworthy that L2–L4 belong to the right side of the bridge, while L5–L7 belong to the left side of the bridge. Observing from the left and right sides, both the overall vehicle flow and the flow of heavy vehicles are higher on the left side, indicating a greater impact of vehicle loads on the left side of the bridge. Among them, the flow of heavy vehicles on L7 is higher than all other lanes, thus requiring particular attention to the safety status of the corresponding box girder of L7.

**Figure 4:**
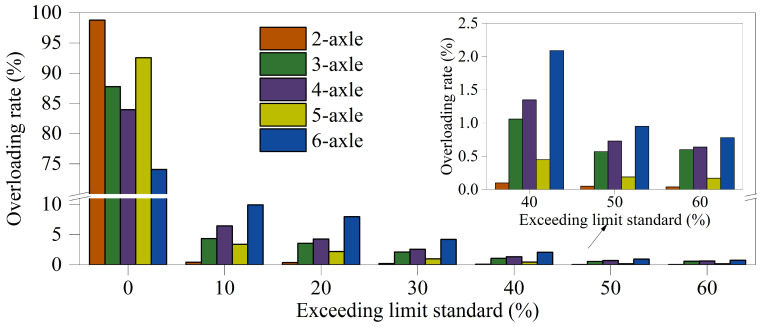
The overloading rate for the representative vehicle types.

Various vehicle operating conditions, including empty, fully loaded, and overloaded states, exert distinctly different effects on small- and medium-span bridges. Overloaded vehicles may force bridges to operate beyond their designed load-carrying capacity, thereby reducing structural reliability and increasing the risk of sudden structural failure. According to the latest “Regulations on the Management of Overlimit Transport Vehicles on Highways,” overloading is defined for 2-axle, 3-axle, 4-axle, 5-axle, and 6-axle vehicles when their loads exceed 18 t, 27 t, 36 t, 43 t, and 49 t, respectively. Figure 4 presents the statistical proportions of overloaded operations for different vehicle types on the Lieshihe bridge. The results reveal that 98.79% of 2-axle vehicles comply with the prescribed load limits, with only a negligible number of overloading cases, which is primarily attributed to the prevalence of 2-axle private vehicles as opposed to freight vehicles. In contrast, the proportion of overloaded vehicles in the remaining four categories ranges from 10% to 30%, with a particularly high overloading rate observed for 6-axle vehicles.

### 4.2. Vehicle Weight Statistics

**Figure 5:**
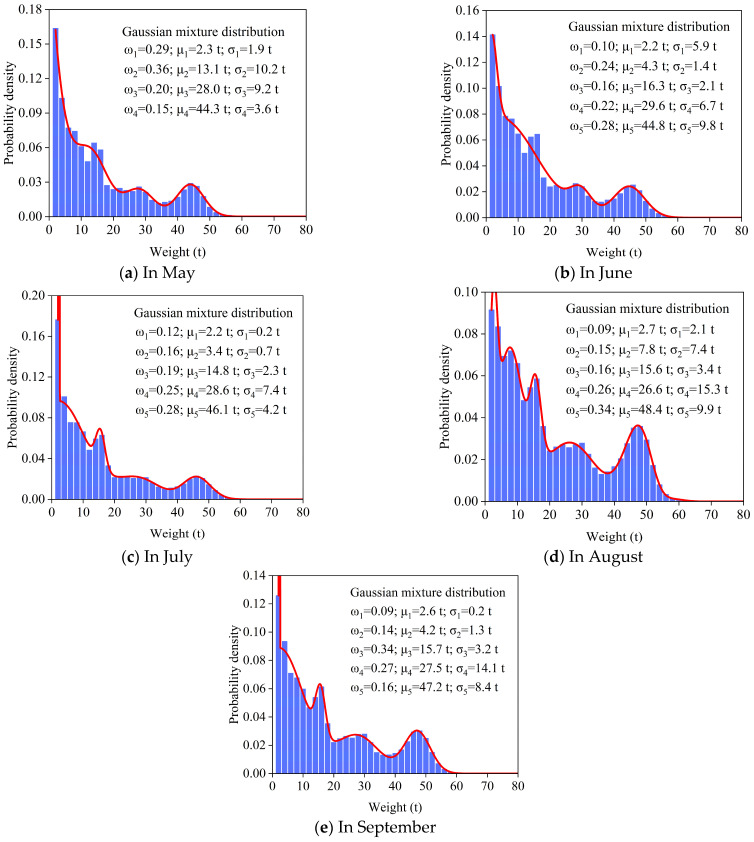
The probability distribution function of vehicle weight in L6 for each month.

Vehicle weight is the main dynamic load during the operation period of highways and bridges and plays a crucial role in vehicle load modeling. Temporal and spatial variations in vehicle loads exert significant influences on highway and bridge structures, which are mainly manifested in the following aspects: (1) fluctuations in vehicle density and weight at different time intervals; (2) variances in load distribution across lanes due to vehicle selection in different spatial zones. Based on the WIM data collected from May to September 2021, statistical models of vehicle weight distributions were established for each traffic lane. L6 is taken as an example to describe the process of establishing the vehicle weight statistical model, and the approach for other lanes is consistent with L6. Figure 5 illustrates that the probability density distribution function of the vehicle weight exhibits a multi-peak characteristic in each month. It can be clearly seen that vehicle weights during bridge operation follow Gaussian mixture distributions. Specifically, the probability distribution function of vehicle weight in May consists of four Gaussian components with different weighting coefficients, whereas those in the remaining months are composed of five Gaussian components with varying weights. The results indicate that the vehicle load information in lane 6 is complex, and that there is considerable variation in the types of vehicle weights during its operational period.

**Figure 6:**
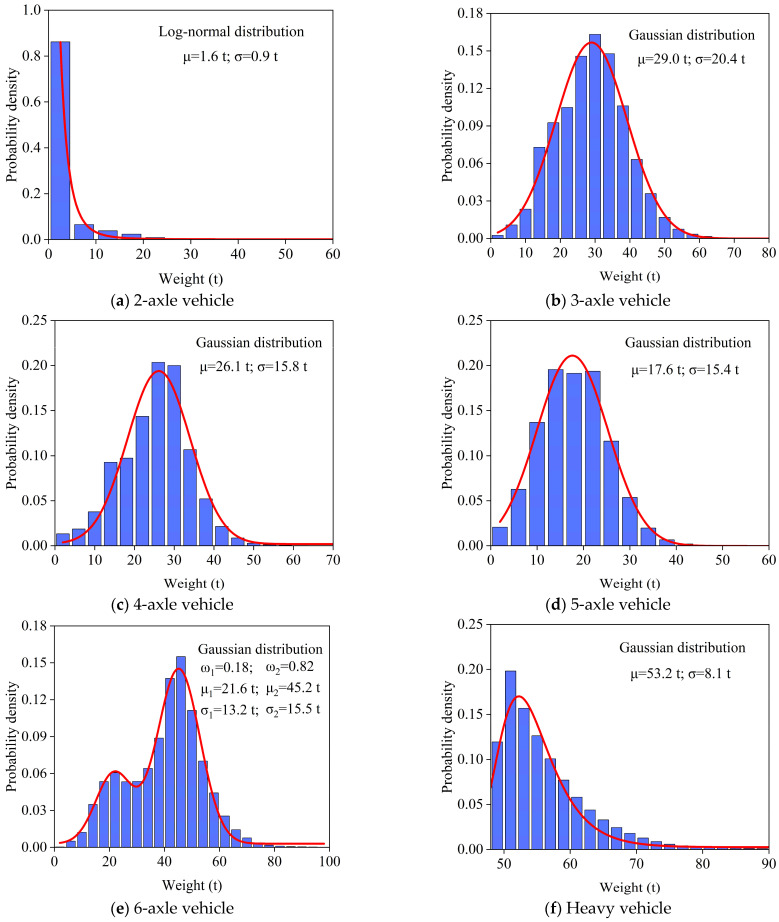
The probability density functions of weight for different vehicle types.

The distribution of vehicle weight also has a significant impact on stochastic vehicle load modeling. Therefore, a statistical analysis was conducted on the weight distributions of the five representative vehicle types and heavy vehicles (>49 t) for the period from May to September in this WIM system. The frequency histogram and probability distribution functions are shown in Figure 6. The results indicate that the probability density functions of the representative vehicle types and heavy vehicles differ significantly in terms of both weight ranges and mean values. These probability density functions accurately capture the statistical patterns of lane-specific vehicle weights on the Lieshihe bridge. Specifically, 2-axle vehicle weights follow a log-normal distribution, mainly concentrated around 1.6 t, with a maximum weight of 47.56 t; the weight of 3-axle vehicles follows a Gaussian distribution, primarily concentrated around 29 t, with a maximum weight of 59.61 t; the weight of 4-axle vehicles is predominantly centered around 21.6 t, with a maximum weight of 69.4 t; the weight of 5-axle vehicles is concentrated around 17.6 t, with a maximum weight of 79.82 t; and the weight of heavy vehicles is mainly concentrated around 53.2 t. It should be noted that the weight of 6-axle vehicles follows a Gaussian mixture distribution, mainly concentrated around 21.6 t and 45.2 t, with a maximum weight of 99.97 t. This indicates that the weight information of 6-axle vehicles varies greatly and conforms to a multimodal distribution.

### 4.3. Axle Load Statistics

**Figure 7:**
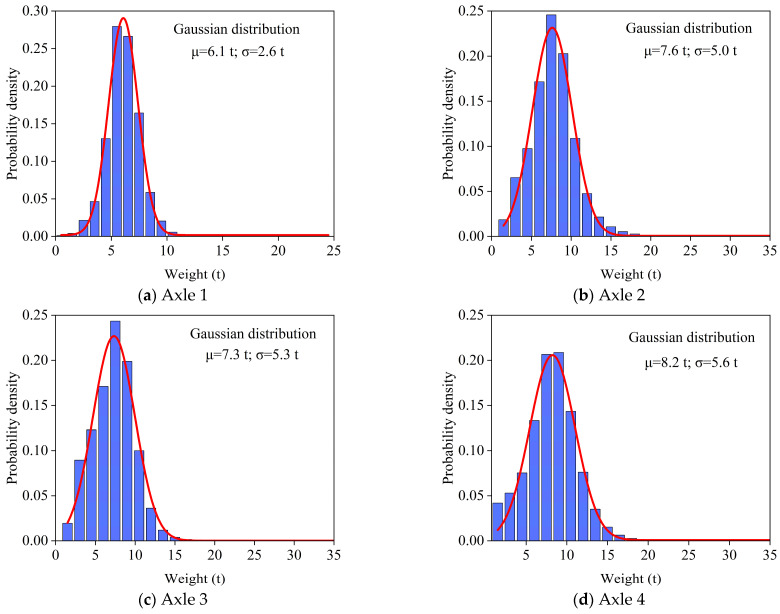

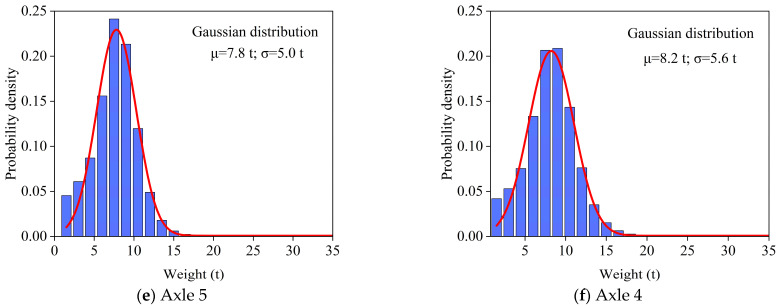
The probability density functions of axle load for 6-axle vehicles.

To accurately establish the stochastic vehicle load model of the Lieshihe bridge, a statistical analysis was conducted on the axle loads of representative vehicle types in the WIM monitoring data, and the probability density functions of axle load for the representative vehicle types were established for each lane. Taking 6-axle vehicles on L7 as an example, the process of establishing the axle load statistical model is explained, and the probability density functions of the representative vehicle types in other lanes is consistent with that of the 6-axle vehicle. Figure 7 shows the frequency bar chart of axle loads for a 6-axle vehicle on L7. A Gaussian distribution was selected to fit the data, resulting in unimodal probability density functions for each axle. Notably, the axle loads for each axle of the 6-axle vehicle are predominantly centered around specific values: 6 t for axle 1, 7.6 t for axle 2, 7.3 t for axle 3, 8.2 t for axle 4, 7.8 t for axle 5, and also 7.8 t for axle 6. The main reason for the differences in axle load distribution of the same vehicle type lies in the different axle group configurations and load conditions. Furthermore, the axle loads of the 6-axle vehicles in L7 from May to September all follow a unimodal Gaussian distribution, which can reflect the statistical regularities of axle loads for various vehicle types on the Lieshihe bridge.

### 4.4. Vehicle Speed Statistics

**Figure 8:**
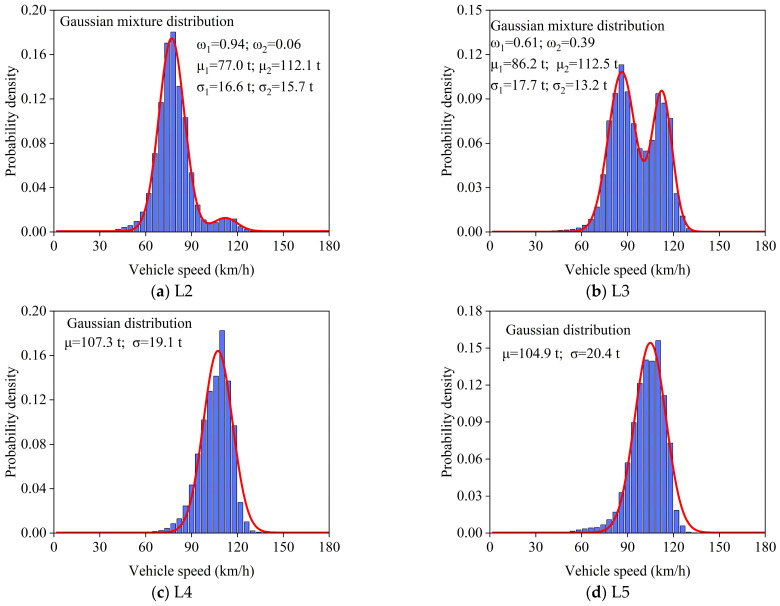

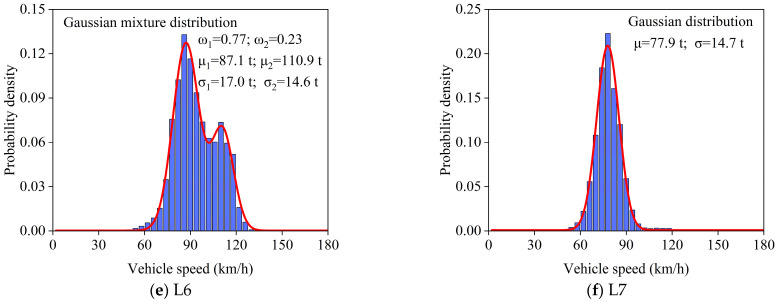
The probability density functions of vehicle speed for each lane.

Vehicle speed is one of the important parameters in the operation status of vehicle loads, which has a certain impact on the driving distance and impact coefficient. Therefore, it is necessary to conduct statistical analysis on the vehicle speed from the WIM measured data to obtain a probability distribution model of vehicle speed to simulate the actual operation of stochastic vehicle loads. Vehicle speeds are subject to variations across different time periods and traffic lanes. Figure 8 shows the frequency histograms of vehicle speed in each lane and establishes the corresponding probability distribution model. The results show that speed does not necessarily follow a unimodal distribution. The speeds of L4 and L5 for fast vehicles exhibit a unimodal Gaussian distribution, with the speed of L4 around 113.2 km/h and lane 5 around 111.4 km/h. The speed of slow L7 also follows a unimodal Gaussian distribution, with the speed around 77.4 km/h. In addition, the speed distribution of lanes 2, 3, and 6 shows a Gaussian mixture distribution, with the speed of L2 around 80 km/h and 112.6 km/h, lane 3 around 87.4 km/h and 114.5 km/h, and L6 around 88.6 km/h and 110.5 km/h, indicating serious overspeed issues. Furthermore, the speed distribution of L2, L3, and L6 is more divergent, mainly due to the mixing of various vehicle types on these lanes.

### 4.5. Results of the Proposed Stochastic Vehicle Load

**Figure 9:**
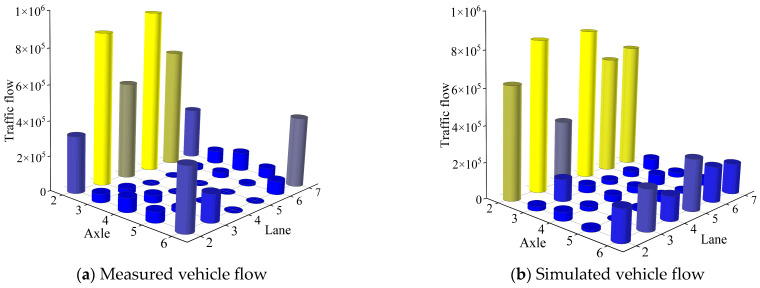
Vehicle flow of each lane for each vehicle type.

The above established probabilistic distribution model of vehicle load characteristic parameters are utilized for random sampling from the known probability distribution functions to generate stochastic vehicle load samples. Figure 9 displays the simulated stochastic vehicle load samples for each traffic lane of the Lieshihe bridge. The spatial distribution of the proposed stochastic vehicle loads exhibits good agreement with the measured samples obtained from the WIM system. Nevertheless, the proposed stochastic vehicle load model slightly overestimates the actual traffic volume of 2-axle and 6-axle vehicles. Notably, L2 and L7 are designated as slow lanes, with WIM measurements indicating predominant traffic by 6-axle and 2-axle vehicles. Surprisingly, the proposed stochastic vehicle load model underestimates the traffic volume of 6-axle vehicles in these lanes while it overestimates the quantity of 2-axle vehicles.

**Figure 10:**
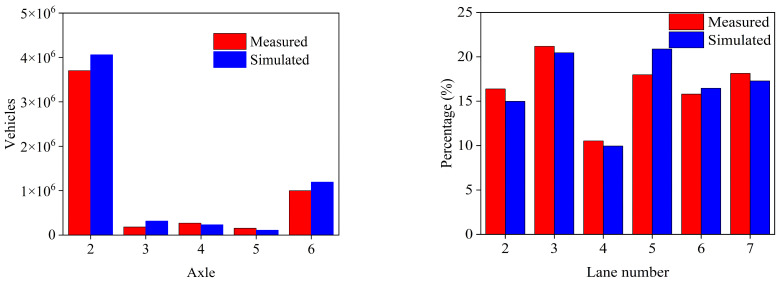
Comparison of vehicle types and lane flow in simulation and measured.

The success of the simulation relies on the degree of agreement between the distribution characteristics of the Monte Carlo-generated stochastic vehicle load samples and the actual measurements obtained from the WIM system. To comprehensively evaluate the consistency between the simulated stochastic vehicle load and the WIM data, further comparative assessments were conducted on various vehicle load parameters. Subsequently, the simulated samples of vehicle types and lane flow volumes are compared with the corresponding measured data, as shown in Figure 10. The results demonstrate a strong agreement between the vehicle type distributions and lane traffic volumes generated by the proposed method and those observed in the WIM measurements. Minor discrepancies are observed in the sample sizes of the simulated 2-axle, 3-axle, and 6-axle vehicles compared with the measured samples, indicating a conservative approach to design. Additionally, the samples of the proposed approach for L5 exhibit a substantial overestimation compared to the measured data, resulting in a traffic flow error of 4.1%. The analysis demonstrates that the proposed stochastic vehicle load model with a sample size of 2.64 million satisfies the engineering technical requirements.

## 5. Conclusions

Based on the vehicle load collected from the WIM system at Lieshihe bridge, more than 2.6 million vehicle records were subjected to statistical analysis. The critical parameters for vehicle load operation included vehicle weight, type, axle load, speed, and number of overloads. Based on multiple vehicle characteristic parameters, the measured vehicle loads were classified into five representative vehicle types for statistical analysis. Various probability distribution models were employed to fit the statistical results, and the corresponding probabilistic models of vehicle load characteristics were established. On this basis, an effective stochastic vehicle load model was developed using Monte Carlo random sampling. The main conclusions are summarized as follows:

(1) Statistical analysis of vehicle flow and overloading conditions in each lane of the bridge shows that vehicle flow from May to September exhibits an initial increasing trend followed by stabilization. The traffic volume in the left lane is higher than that in the right lane. Two-axle vehicles rarely experience overloading, whereas 3-axle, 4-axle, and 6-axle vehicles exhibit pronounced overloading behavior, with a relatively high percentage of heavy vehicles in L7.
(2) The vehicle weights in each lane exhibit strong stochastic characteristics. The 2-axle vehicle weight mainly follows a log-normal distribution, with most weights below 5 t. The weights of 3-axle, 4-axle, and 5-axle vehicles follow unimodal Gaussian distributions, while the weight of 6-axle vehicles follows a bimodal Gaussian distribution. The proportion of heavy vehicles is higher for 3-axle and 4-axle vehicles compared to 5-axle vehicles. Axle loads of all vehicle types follow unimodal Gaussian distribution, with a uniform axle load distribution.
(3) The vehicle speed distribution characteristics vary significantly among different lanes and can be mainly described by unimodal and bimodal Gaussian models. Lanes L4 and L5, which exhibit higher operating speeds, present unimodal Gaussian speed distributions, with speeds around 113.2 km/h and 111.4 km/h, and are mainly consisting of 2-axle vehicles traveling at high speeds. Speed distributions in L2, L3, and L6 follow Gaussian mixture distributions, whereas L7, dominated by heavy vehicles, exhibits a unimodal Gaussian distribution. Over long-term operation, vehicle speed distributions in each lane remain relatively stable.
(4) The Monte Carlo-based stochastic vehicle loads match well with the measured stochastic vehicle loads. The simulated samples exhibit the same distribution characteristics as the WIM measured data, thereby satisfying practical engineering and technical requirements.

The establishment of the stochastic vehicle load model and the proposed simulation methodology enables the realistic reproduction of the actual operating conditions of vehicles traversing the bridge once representative vehicle sample data are available. On this basis, the work can provide essential technical support for subsequent analyses of the dynamic responses of small- and medium-span bridges subjected to vehicle loads, especially for urban bridges. Future research will further focus on evaluating the structural safety conditions and remaining service life of the bridge.

## Figures and Tables

**Table 1:** Vehicle flow count for each lane of the bridge.

Month	Lane	Vehicle Flow	Weight > 3 t	Ratio (%)	Weight > 49 t	Ratio (%)	Vehicles (>49 t)	Vehicles (>3 t)
May	L2	156,916	133,851	85.3%	10,400	6.63%	49,752	442,257
	L3	180,276	67,886	37.66%	201	0.11%		
	L4	214,023	2964	1.38%	17	0.007%		
	L5	268,737	1982	0.74%	0	0		
	L6	248,706	87,485	35.17%	1793	0.72%		
	L7	161,656	148,089	91.6%	37,341	23.1%		
June	L2	1,964,644	166,562	85.57%	17,382	8.93%	72,250	545,195
	L3	230,377	81,842	35.52%	273	0.11%		
	L4	198,586	2565	1.29%	3	0.0015%		
	L5	327,154	2993	0.91%	1	0.0003%		
	L6	280,464	111,878	39.89%	3534	1.26%		
	L7	190,507	179,356	94.14%	51,057	26.8%		
July	L2	193,403	163,281	84.42%	17,226	0.89%	52,313	563,848
	L3	279,752	97,036	34.68%	6737	2.4%		
	L4	145,621	1841	1.26%	1	0.00068%		
	L5	357,712	3666	1.02%	11	0.00307%		
	L6	307,912	107,565	34.93%	4099	1.33%		
	L7	204,249	190,459	93.24%	24,239	11.86%		
August	L2	158,746	145,848	91.87%	14,980	9.43%	58,748	536,630
	L3	187,939	98,331	52.32%	9006	4.79%		
	L4	6	0	0	0	0		
	L5	254,775	3115	1.22%	4	0.00157%		
	L6	212,143	115,364	54.38%	7720	3.63%		
	L7	179,951	173,972	96.67%	27,038	15.02%		
September	L2	164,316	134,945	82.12%	8017	0.48%	42,867	561,837
	L3	245,390	102,349	41.7%	8296	3.38%		
	L4	3	0	0	0	0		
	L5	320,759	3562	1.11%	3	0.00093%		
	L6	284,292	129,593	45.58%	7911	2.78%		
	L7	202,046	191,388	94.72%	18,640	9.22%		
May to September	L2	2,638,025	744,487	28.22%	68,005	2.58%	275,930	2,649,767
	L3	1,123,734	447,444	39.81%	24,513	2.18%		
	L4	558,239	7370	1.32%	21	0.04%		
	L5	1,529,137	15,318	1.00%	19	0.01%		
	L6	1,333,517	551,885	41.39%	25,057	1.88%		
	L7	938,409	883,264	94.12%	158,315	16.87%		

## Data Availability

The original contributions presented in this study are included in the article. Further inquiries can be directed to the corresponding authors.
